# The usefulness of elastography in the evaluation and management of adult men with varicocele: A systematic review

**DOI:** 10.1080/2090598X.2021.1964256

**Published:** 2021-09-16

**Authors:** Jibril Oyekunle Bello, Kamran Hassan Bhatti, Nazim Gherabi, Joseph Philipraj, Yash Narayan, Georgios Tsampoukas, Nisar Shaikh, Athanasios Papatsoris, Mohamad Moussa, Noor Buchholz

**Affiliations:** a U-merge Ltd. (Urology for Emerging Countries), London, UK; b Urology unit, Department of Surgery, University of Ilorin Teaching Hospital, Ilorin, Nigeria; c Urology section Hamad Medical Corporation Alkhor Qatar; d Andrology committee of the Algerian Association of Urology, Algiers, Algeria; e Department of Urology, Mahatma Gandhi Medical College and Research Institute, Sri Balaji Vidyapeeth, Puducherry, India; f Department of Urology, The Princess Alexandra Hospital NHS Trust, Harlow, UK; g Department of Urology, Shaheed Mohtarma Benazir Bhutto Medical University Larkana, Larkana, Pakistan; h 2nd Department of Urology, School of Medicine, Sismanoglio Hospital, National and Kapodistrian University of Athens, Athens, Greece; i Department of Urology, Al Zahraa Hospital, University Medical Center, Lebanese University, Beirut, Lebanon

**Keywords:** Elastography, varicocele, stiffness, male infertility

## Abstract

**Objective:**

To review the role of elastography in the evaluation and decision-making of adult, infertile men with varicocele.

**Methods:**

A systematic search using the terms (Elastography) AND (Varicocele), (Stiffness) AND (Varicocele), (Elastography) AND (Male infertility) was performed in Pubmed/Medline. Studies reporting a) elastographic characteristics in varicocele-bearing comparing to normal testicles, and b) the correlation of elastography with varicocele grading, parameters of spermatogenesis, and outcomes of varicocele treatment were selected. Exclusion criteria were animal, adolescents, abstracts, and non-English language studies.

**Results:**

In total, 453 articles were identified; 11 eligible studies were selected. Several modalities were used (shear wave elastography, strain elastography, quasistatic ultrasound elastography, acoustic radiation force impulse). Varicocele-bearing testicles have significantly different stiffness and elasticity in comparison to normal and non-varicocele testicles. Although not in full agreement, elastography readings are correlated with semen parameters. Conflicting results were reported regarding grading as most of the studies failed to demonstrate a significant correlation. Shear wave elastography showed a significant correlation with the improvement in semen parameters after varicocelectomy, but the association with pregnancy rates is unknown. Finally, no studies were identified comparing elastography with other modalities.

**Conclusions:**

Elastography can detect changes in the architecture of varicocele-bearing testicles. Although the role of the modality in grading is uncertain, elastography showed a meaningful correlation with spermatogenesis parameters. Importantly, elastography readings could predict the improvement in semen parameters after varicocelectomy which is useful in terms of decision-making in infertile men with varicocele.

**Abbreviations:**

ARFI: acoustic radiation force impulse; CDUS: colour Doppler ultrasonography; DWI: diffusion-weighted imaging; PRISMA: Preferred Reporting Items for Systematic Reviews and Meta-Analyses; SWE: shear wave elastography; VC: varicocele

## Introduction

Varicocele (VC) is defined as the ‘abnormal dilatation of the pampiniform plexus in the spermatic cord’. A clinical VC can be seen in 15% of the normal male population, while it might be involved in up to 40% and 80% of primary and secondary infertility patients, respectively [1]. Pathophysiologically, VC alters the blood flow in the testes provoking a cascade of oxidative stress phenomena that can finally result in testicular damage and possible subfertility [2].

Although the co-existence of infertility and VC is considered a strong indication for treatment, not all patients will enjoy fatherhood after intervention [3]. This highlights the necessity of proper patients’ selection and modern practice has explored the role of various imaging modalities for the optimal management of VC. Grey-scale and conventional colour Doppler ultrasonography (CDUS) facilitate the diagnosis, whereas they contribute partially to the evaluation of the effect on spermatogenesis and the outcome of treatments, utilising parameters such as the vein size, the type of reflux and the intratesticular haemodynamics [4–6]. Other novel modalities such as MRI and multiparametric CDUS appear more promising as they can evaluate the degree of testicular interstitial fibrosis and architecture, which is can reflect the possible damage caused by the VC [7]. However, the interpretation of the US findings has not been standardised, whereas the modality is not fully decisive in the decision-making. Moreover, although MRI looks promising and offers an objective evaluation of the testicular parenchyma, the greater limitation lies in the cost, availability and experience of the operator.

Ultrasonic elastography is a novel ultrasonic modality which has been previously used in male infertility and for the investigation of scrotal and prostate pathologies [8–10]. There are two basic elastography approaches. Strain elastography measures the longitudinal tissue displacement before and after compression, usually by manual manipulation of the ultrasound transducer, providing an indication of relative stiffness of an area of interest compared to its surroundings. Using shear wave elastography (SWE), shear waves are generated by repetitive compression produced by high-intensity pulses from the ultrasound transducer, which allows a more quantitative estimate of tissue stiffness [11]. Early elastography results in patients with VC have shown a significant correlation between testicular elasticity and grade in infertile men with dyspermia, broadening the potential of the modality in these patients [12]. Elastography could be able to combine an affordable cost along with increased objectivity, allowing reproducibility in the interpretation of the findings and facilitating the patients’ selection for treatment.

To the best of our knowledge, no previous cumulative data have been presented regarding the role of the modality in men with VC. In the present literature review, we investigated the role and usefulness of elastography in the evaluation and management of adult men with VC.

## Methods

**Figure 1.**
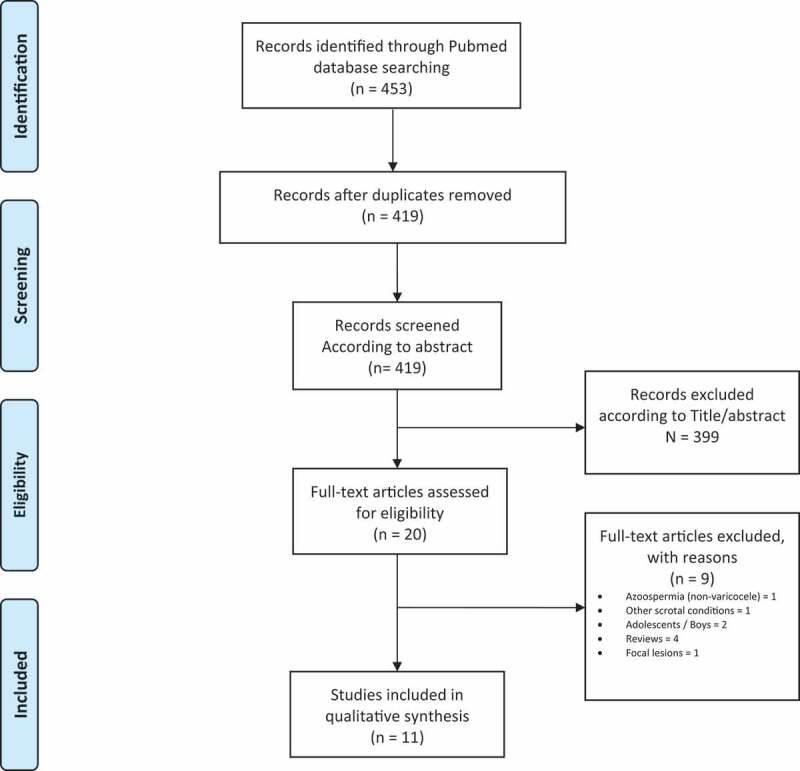
PRISMA flow diagram of search strategy

A systematic search according to the Preferred Reporting Items for Systematic Reviews and Meta-Analyses (PRISMA) Guidelines was performed on PubMed/MEDLINE [13]. No time limitations or type of studies (prospective, retrospective) were applied. We used the terms (Elastography) AND (Varicocele), (Stiffness) AND (Varicocele), (Elastography) AND (Male infertility), and an advanced search using the terms (ultrasound [Title/Abstract]) AND (varicocele [Title/Abstract]) was performed searching for additional results. Inclusion criteria were studies reporting observations on and application of elastography in adult men with VC. Exclusion criteria were studies on animals, adolescents, abstracts, and non-English language studies. The studies were selected based on the follow criteria: a) reports on stiffness characteristics in testicles with VC, b) the correlation of elastography with VC grading (clinical or sonographic), c) the correlation of elastography with parameters of spermatogenesis (hormones, semen parameters, testicular volume, or pregnancy rates), and d) the role of elastography as predictor of VC treatment (improvement in semen parameters or pregnancy rates). Our strategy search is illustrated in Figure 1. The data extracted included: publication year, age, number of participants, indication, modality used, study design, fertility status, aim and primary and secondary outcomes of the studies relevant to our principal scientific question.

## Results

The search engine provided 453 articles for evaluation. After the removal of duplicates, 419 articles were screened through Title/Abstract and 20 full-text articles were examined for their eligibility. Finally, 11 articles were identified meeting the search criteria. All trials were prospective, whereas no studies were identified comparing or correlating elastography with other modalities. The characteristics of the studies are presented in Table 1 [8,12,14–22]. In Tables 2–5 we present and comment on the studies related to our scientific questions.

**Table 1.** Studies characteristics

	Modality	Material and methods	Aims of the study
Dede et al. [12]	ARFI elastography	Prospective Study 30 patients with left VC and infertility (mean [SD] age 29 [7.8] years). 30 normal controls (mean [SD] age 27 [0.64] years)	To assess the impact of VCs on testes using elastography To correlate elastography results with patient’s hormone levels and semen analysis parameters
Zeng et al. [20]	Quasistatic ultrasound elastography	Prospective study 1073 patients were evaluated for scrotal pathology due to pain Age 10–75 years, mean [SD] 52 [11.73] years Of the 262 patients diagnosed with clinical VC, 201 had left-sided VC only, 19 right-sided only, and 42 had bilateral VC	To assess the value of using quasistatic ultrasound elastography in the evaluation of scrotal lesions To describe the features of normal testes and scrotal lesions on quasistatic elastography
Abdelwahab et al. [22]	SWE	Prospective study 47 patients (1 was excluded due to lost in follow-up) Mean [SD] age 30.89 [3.77] years Testicular SWE before subinguinal microsurgical VC ligation Semen analysis performed before and 6 months after varicocelectomy	To investigate the role of preoperative SWE as a predictor for improvement in semen analysis in patients with primary infertility and clinically detectable VC
Küçükdurmaz et al. [8]	Strain elastography	Prospective study 61 patients with a diagnosis of primary infertility were evaluated Patients were divided into two groups based on semen analyses, normal (Group 1, *n* = 31, mean [SD] age 33.39 [6.45] years) and abnormal (Group 2, *n* = 30, mean [SD] age 34.3 [6.7] years) VC was present in 11 men in Group 1 and 12 men in Group 2	To evaluate the diagnostic value of strain elastography in the assessment of infertile patients To investigate the correlation between strain elastography of testicular tissues with hormone levels and semen analysis
Rocher et al. [14]	SWE	Prospective Study 601 patients (62 normal, mean [SD] age 37.9 [14.5] years, and 539 with proven infertility). Of the 539, 349 (mean [SD] age 36.7 [7.6] years) had OAT; 132 men had left VC	To evaluate the reproducibility and practicality of using testicular SWE To evaluate tissue stiffness in normal and infertile patients and to assess the correlation between testicular volume and stiffness
Yavuz et al. [21]	SWE – ARFI	Prospective study 100 patients, 36 with VC Age 19–49 years, mean [SD] 28.77 [6.11] years	To assess the reliability of testicular stiffness quantification using SWE in evaluating male fertility potential and for pre-diagnosis of diseases through sperm quantification
Bitkin et al. [19]	Strain elastography	Prospective study 30 infertile patients with VC (mean [SD] age 25.61 [6.06] years). 30 normal controls (mean [SD] age 27.94 [4.22] years)	To use strain elastography to evaluate structural testicular changes that occur secondary to VC To assess the relationship between strain elastography and patients’ hormone levels and semen analysis
Salama et al. [18]	Strain elastography	Prospective study 50 infertile men with left VC (mean [SD] age 29.3 [4.4] years) and 20 age-matched controls (mean [SD] age 29.5 [4.6] years)	To use real-time strain elastography to assess testes in patients with VC and to correlate these results with their clinical, hormonal and seminal profiles
Erdogan et al. [16]	SWE	Prospective study 48 patients (mean [SD] age 28.56 [8.95] years) with VC and 52 controls (mean [SD] age 28.79 [11.62] years) were divided into 3 groups (A: testicles with VC, B: contralateral normal testicles, C: normal group)	To use SWE, with measurement of elasticity and volume, to determine histological damage in patients with VC
Fuschi et al. [17]	SWE	Prospective study 82 male patients (mean [SD] age 27.44 [6.095] years) with clinical, left VC and a progressive alteration of semen quality were enrolled Patients were evaluated before varicocelectomy, and at 3 and 6 months postoperatively	To use SWE to evaluate the impact of varicocelectomy on degree of fibrosis, and testes elasticity and function To evaluate the relationship between SWE and patients’ histology and semen parameters
Turna and Aybar [15]	SWE	Prospective study 58 patients (mean [SD] age 32.81 [9.07] years) with left-sided VC and 58 normal controls (mean [SD] age 34.23 [9.09] years) VC group: Patients’ testes were classified into Group A (normospermic) or Group B (oligospermic) Mean SWE values and testicular volume were recorded	To assess the role of SWE to evaluate testes in patients with VC To evaluate the correlation between testicular stiffness and VC

**Table 2.** Elastography values in men with VC

	Modality	Outcome	Comments
Dede et al. [12]	ARFI elastography	Mean elastography results (displacement) were significantly lower in patients who had VCs implying stiffness	Participants were mildly oligospermic (10–15 millions/mL) which underlying pathology is unknown
Zeng et al. [20]	Quasistatic ultrasound elastography	Elastographic results were not statistically different between VC bearing and normal contralateral testicles; the strain ratio did not differ between VC and normal men	The authors did not correlate with fatherhood/fertility Effect of VC on pain is unknown
Küçükdurmaz et al. [8]	Strain elastography	Mean strain ratios but not mean strain values were significantly lower in men with normal semen parameters	Authors stated that statistical analysis showed no effect of presence of VC in elastography findings Number of VC participants was small; 11 and 12 in Group 1 and Group 2, respectively
Rocher et al. [14]	SWE	Median testicular stiffness was significantly lower in patients with a left VC in comparison to normal control group	No significant difference with the contralateral testis; participants had OAT which reflects testicular damage in both testicles
Bitkin et al. [19]	Strain elastography	Strain ratios in the patients with unilateral VC were significantly lower than the control group	Authors stated that elastography might be more sensitive for detecting damage of testicular tissue comparing to testicular size; future histopathological correlation is needed
Salama et al. [18]	Strain elastography	Strain ratio and elasticity score were significantly higher in the VC-affected group	Unknown histopathological correlation with elastography readings
Erdogan et al. [16]	SWE	SWE values were significantly higher between VC-affected, contralateral and normal-control testicles No difference between contralateral and normal-control testicles	Authors did not state fatherhood/fertility status SWE measurements were only taken in single plane
Fuschi et al. [17]	SWE	SWE readings found significantly higher in left, VC-affected in comparison to normal testicles	Absence of control group; stiffness was compared to contralateral testicle
Turna and Aybar [15]	SWE	VC-affected testes were significantly stiffer in comparison to the contralateral testes in both dyspermic and normospermic men The mean SWE value of the left testes in dyspermic men with VC was significantly higher in comparison to the normospermic men with VC	Absence of interobserver variability; lack of histopathological correlation

**Table 3.** Correlation with parameters of spermatogenesis

	Modality	Outcome	Comments
Dede et al. [12]	ARFI elastography	Statistically significant negative correlations between FSH and elasticity	No significant correlation of elasticity with semen parameters (sperm count, motility, morphology) Only patients with mild oligospermia were evaluated
Küçükdurmaz et al. (2017) [8]	Strain elastography	Significant correlation of elastography with sperm count, total sperm count, total motile sperm count and morphology in men with dyspermia including men with VC Multivariate analysis between FSH and elastographic findings revealed no significant correlation in VC patients	Patients with VC were a small proportion of the study Strain ratios were found to be positively correlated with testicular volume in normospermic but not in dyspermic men
Yavuz et al. [21]	SWE – ARFI	Significant negative correlation between mean testicular shear wave velocity values and sperm counts and testicular volume Highest mean shear wave velocity values found in the azoospermia group and lowest in normospermia group	Hormonal profile was not assessed Cut-off values to differentiate between groups showed low sensitivity and specificity
Bitkin et al. [19]	Strain elastography	No significant relationship was found between the left testicle strain ratio and the seminal parameters, hormonal profile and the left testicular volume in the VC group	Main group had mild oligospermia which might reflect the negative correlations
Salama et al. [18]	Strain elastography	Significant negative correlation between elasticity score and the testicular volume and the percentage of normal forms No correlation between hormonal profiles and elastographic parameters	Lack of histopathological correlation
Erdogan et al. [16]	SWE	Although testicular volume was significantly different between groups, no significant correlation was observed between the testicular volume and SWE in all groups	Fertility status not documented No correlation with histopathological findings, hormonal profiles, or semen parameters was examined
Turna and Aybar [15]	SWE	No significant correlation was observed between testicular stiffness and testicular volume irrespective of dyspermia	No correlation with semen parameters or hormonal profile was examined A ‘weak’ (*P* = 0.014) negative correlation was detected between the volume of the testes and VC grade

**Table 4.** Correlation with grade

	Modality	Outcome	Comments
Dede et al. [12]	ARFI elastography	A significant negative correlation between VC grade and elasticity of testes	Distribution of grade is not reported Small number of participants
Küçükdurmaz et al. [8]	Strain elastography	No effect of grade of VC on elastography findings	Number of VC participants was small
Yavuz et al. [21]	SWE – ARFI	No relationship between the presence or the grade of VC and the mean shear wave velocity values of testes	Patients with VC were a small portion of the study Four heterogenic groups (azoospermia, oligozoospermia, isolated asthenospermia and complete normospermia)
Salama et al. [18]	Strain elastography	VC grade showed significant positive correlations with both the strain ratio and elasticity score	Process of elastographic imaging was not completely blinded as the operator was aware of the VC grade when performing the testicular assessment
Turna and Aybar [15]	SWE	No correlation was observed between testicular stiffness and VC grade	Weak but significant negative correlation was detected between the volume of the testes and VC grade

**Table 5.** Prediction of treatment outcome

	Modality	Outcome	Comments
Abdelwahab et al. [22]	SWE	At a cut-off value of 4.5 kPa, the stiffness index showed a sensitivity of 86.4% and a specificity of 84.2% for semen parameter improvement after varicocelectomy Statistically significant negative correlation between SWE stiffness index and both sperm count and total motility improvement, but not for morphology	Absence of control group with normal testicular stiffness value and histopathological correlation The SWE reading were not repeated postoperatively No reporting of pregnancy rates
Fuschi et al. [17]	SWE	A significant negative correlation between postoperative SWE of left testis and ipsilateral testicular volume and sperm count at 3 months but not for morphology 6-months postoperative testicular biopsies revealed a morphological recovery with significant disappearance of epithelium thickening, apoptosis and vacuolisation No correlation between improvement in stiffness and change in hormonal levels (FSH, LH, testosterone) pre- and postoperatively	The indication for treatment was made due to deterioration in semen quality – no reporting of infertility status No reporting of pregnancy rates

### Testicular stiffness in VC- in comparison to non-VC-bearing testicles and normal controls

In a study using acoustic radiation force impulse (ARFI) elastography, the authors found that all elastography measurements (upper, middle, lower pole and mean readings) were significantly lower in the VC-bearing testicles (oligospermic men with various grades of clinical VC) in comparison to the left testicles of normospermic men with no VC [12]. The testicular stiffness measure with SWE of the VC-bearing testicles in infertile men with unilateral clinical or subclinical VC and oligo-astheno-teratozoospermia (OAT) was found to be significantly lower in comparison to fertile men and normal testes [14]. Turna and Aybar [15] found that VC testicles were stiffer in comparison to the contralateral normal ones in patients who were normospermic or oligospermic, whereas testicles with VC were stiffer in comparison to testicles of normal controls regardless of dyspermia; on the other hand, there was no difference in stiffness between the right and left testicles of normal controls. Higher SWE values in VC-bearing testicles in comparison to the contralateral normal ones were also seen in a recent study but the fertility status of the participants was not documented [16]. Finally, a recent prospective study of men with Grade III VC and worsening semen parameters reported significantly higher SWE values in the left testicles carrying VC in comparison to the right side [17].

Using real-time strain elastography, Salama et al. [18] reported a significantly higher strain ratio and elasticity scores in infertile men with VC in comparison to normal controls, whereas in a second study, the left testicle strain ratio median value was found to be significantly lower in oligospermic men in comparison to a normal group [19]. In a study by Küçükdurmaz et al. [8], dyspermic men including men with VC had different strain ratio readings in comparison to controls but the authors after sub-analysis concluded that the presence of VC did not have effect on elastography findings. Finally, in men being evaluated with strain elastography for various scrotal pathology, elastograms between VC-bearing testicles and normal controls were similar and the strain ratios were not significantly different [20].

### Correlation of stiffness with markers of spermatogenesis

The landmark study of Dede et al. [12] showed a significant negative correlation between FSH and elasticity, which might directly reflect the subsequent testicular impairment. Although in the same study there was no correlation between elastography and semen parameters. Using strain elastography, a study showed no correlation with FSH in infertile men with VC (with dyspermia or normospermia), although the trial showed significant correlations of strain values with total motile sperm count and sperm morphology in infertile men with dyspermia (including men with VC) [8]. In another study, no significant relationship between the left testicular strain ratio and the seminal parameters, hormonal values and the left testicular volume was found in oligospermic infertile men with VC [19]. Finally, Salama et al. [18] reported that the strain ratio and elasticity scores showed a significant negative correlation with sperm morphology, whereas the elasticity score showed a significant negative correlation with testicular volume.

The use of SWE has also shown significant correlations with parameters of spermatogenesis. In 100 men with various degrees of dyspermia (azoospermia, oligospermia, decreased motility and agglutination, complete normospermia) including 36 patients with VC, the mean shear wave velocity values showed strong negative correlations with the mean testicular volume and sperm count [21]. Erdogan et al. [16] noted a significant difference in testicular volume between VC-bearing testicles, contralateral normal testicles, and controls, but no correlation between SWE readings and testicular volume. The authors mentioned that ‘testicular volume is not a reliable parameter for reflecting the degree of parenchymal damage’, but there was no correlation with histopathological findings where the participants were not mentioned to be infertile. Finally, in a study including oligospermic and normospermic men with VC, testicular volume was also lower on the side with VC in comparison to the contralateral normal ones (irrespective of oligospermia or not), but no significant correlation was observed between SWE stiffness and the volume of the testes [15].

### Correlation of stiffness with VC grade

While some studies report a significant correlation of elastography with VC grades, others found no merit. The discrepancies might be related to the modality used, the stage of VC development or the grading system (clinical vs sonographic).

In the study by Dede et al. [12], a significant negative correlation was found between elastography readings and grade, which in practical terms means that the when grade increases, the elasticity decreases. Another study using strain elastography assessed the relationship between clinical grading and VC. The authors found significant differences between the different grades of VC and the elastographic parameters, whereas VC grade showed a significant positive correlation with both the strain ratio and elasticity score [18].

On the other hand, a study found no correlation between the sonographic grade (classified according to the Sarteschi system) and SWE readings in 36 infertile patients with Grade I–III VC [21]. Using the same modality, no correlation was found between clinical grade and SWE readings in a study of 58 men with VC (normospermic and oligospermic) [15]. Finally, in a similar population of patients, a study stated no correlation between grade and strain elastography readings. In this study, the sample was rather small as 23 men with VC and fertility issues were included; 11 men had normal semen analysis whereas 12 men were dyspermic [8].

### Pre- and postoperative elastography as predictor of treatment outcome

Two prospective studies have reported the utility of testicular stiffness relating to outcome after surgical intervention.

The first trial included 48 men with a mean age of 30.9 years, with unilateral clinical VC and mean duration of infertility of 3 years. Using testicular SWE, the authors found that a mean cut-off value of 4.5 kPa predicted efficiently the improvement in semen parameters after microsurgical varicocelectomy. The aforesaid cut-off had a sensitivity of 86.4% and a specificity of 84.2%, and a statistically significant negative correlation between the stiffness index and improvement in sperm count and total motility was found [22].

A second study included 82 men with a mean age of 27.44 years and Grade III–IV VC (according to the Sarteschi system) who had significant deterioration in their semen analysis during follow-up. After a laparoscopic transperitoneal varicocelectomy, the authors noticed that the left testicular volume increased whereas the left SWE decreased, and both parameters showed significant differences compared to baseline. Moreover, a significant positive correlation between the difference of pre- and postoperative left SWE and testicular volume was found. Also, a significant negative correlation between postoperative SWE of left testis and ipsilateral testicular volume and sperm count at 3 months was observed [17].

## Discussion

In summary, elastography was found to be able to differentiate between VC-bearing testicles and the normal ones in most cases. The finding implies that elastography might be able to detect the effect of VC on the testicular parenchyma assisting the physical examination and when assessing the eutrophic or atrophic status of the testicles. Regarding the association of elastography with grading though, reports have been conflicting and it cannot be supported that the modality could replace clinical grading. Although some parameters of spermatogenesis were correlated significantly with some elastography features, the most promising findings were observed regarding the association of SWE and the outcomes of treatment. The evidence that elastography could predict the improvement in semen parameters is significant and it might help the clinician when the most cost-effective modality for the management of infertile men with VC is sought.

Specifically, elastography was reported as a marker of underlying architecture or atrophy and is considered more informative than palpation [12]. When using elastography in infertile men, a clinical correlation with the current clinical context is mandatory though. For example, in their study, Rocher et al. [14] included infertile men with VC and OAT, and reported lower SWE in comparison to controls but no significant difference with the contralateral testis. In contrast, other authors found that the VC testicles were stiffer compared to the contralateral normal testicles regardless of the presence of dyspermia [15]. The discrepancy in the above findings is explicable considering that infertile men with severe damage to spermatogenesis and semen abnormalities may have similar histological changes in both testicles extending up to the degree of testicular failure. Similarly, two studies using strain elastography reported inverse elasticity readings in VC testicles in comparison to controls [18,19]. If infertility is absent, no difference might be found between VC-bearing and normal testicles as reported in one study [20]. Thus, the clinical context has to be considered; discrepancies due to the modality used, and the experience of the operator need to be considered prior to selection of the optimal modality.

For a correlation with spermatogenesis parameters, elastography readings have been shown to be correlated with testicular volume, FSH and semen parameters. In a group of men with dyspermia including men with VC, an inverse relationship between strain values and total motile sperm count has been reported [8]. Total motile sperm count is considered a reliable marker of pregnancy rates and the findings sound meaningful [23]. Whether all these correlations are useful though is uncertain, as in practice, when male infertility has been diagnosed and VC has been implied as the cause, clinical rather than radiological criteria will guide the management [24]. However, elastography might be useful when evaluating newly diagnosed men with VC or post-pubertal, young men not desiring fatherhood at the time of diagnosis. Although the specificity and the sensitivity of the modality must be clarified, the proper use of elastography by experienced radiologists also carries merit when surveillance rather than intervention has been decided. In adolescents, the significant correlations between elastography and testicular volume might assist with the indication for treatment, although the modality does not seem to alter dramatically other traditional clinical criteria used for intervention [25,26].

The clinical classification by Dubin and Amelar remains the cornerstone of diagnosis, but the main drawback is the lack of predictive significance [27]. Therefore, although elastography has shown correlation with clinical grade [12,18], we consider that the practical merit of the correlation of imaging characteristics with clinical grading is uncertain, unless the former ones contribute to the assessment of the severity of the condition relating to spermatogenesis. CDUS systems based on vein size, duration of reflux and testicular volume are useful for the diagnosis and can stratify VCs [4,28–30], whereas individual parameters such as reflux might also draw useful conclusions [31,32]. Multiparametric MRI using dynamic contrast-enhanced MRI, diffusion-weighted imaging (DWI) and MR spectroscopy have been proposed for the stratification of testicular damage induced by the VC, as a significant correlation has been shown between the apparent diffusion coefficient and venous diameter on one hand, and DWI and testicular damage on the other hand [33]. Ultrasound testicular contrast harmonic imaging is also as an adjunctive tool for the assessment of spermatogenesis in VC-bearing testicles [34]. In a similar manner, regardless of the direct association with clinical grading, elastography should be considered useful due to the correlation with parameters of spermatogenesis. This can extend to the assessment of mild or subclinical forms of VC where the data are still conflicting and not supporting an invasive approach except in a select group of patients [35,36].

In terms of treatment outcomes, SWE readings were found to be predictive of the improvement in sperm parameters. Yet, relevant studies did not assess the pregnancy rates after treatment or define semen deterioration as an indication for treatment, which does not necessarily imply infertility. However, elastography might be an adjunctive tool predicting which patients might benefit from meaningful improvements in their semen quality. The latter is important in men undergoing varicocelectomy followed by assisted reproduction techniques [37]. Whether elastography would save unnecessary treatments though remains uncertain. To date, the indications for VC correction are mainly dictated by clinical parameters and varicocelectomy has been proven to be cost-effective if assisted reproduction techniques are to be followed, or even in azoospermic patients [38]. Thus, elastography might not be as decisive and the clinical context will guide the management. However, Fuschi et al. [17] reported that the histopathological reversal of testicular architecture follows the shift in SWE readings, which implies that elastography could document the recovery of spermatogenesis and the success of treatment. Moreover, this might give new insights in unexplored fields, such as bilateral subclinical VCs, where there is no clinical tool apart from a testicular biopsy to assist in the prognosis of men undergoing surgery [39]. Furthermore, elastography could identify sites of healthy testicular tissue and facilitate testicular tissue extraction in men warranting invasive approaches for their fertility treatment, with or without varicocelectomy.

Our present review has some potential limitations that should be appreciated. First, although we attempted to provide a systematic review on the topic, due to the different modalities used and the heterogeneity of the studies, we did not attempt to provide a summary of evidence or recommendations. Similarly, we did not examine the bias accompanying the studies, considering that elastography should be considered experimental in patients with VC and principal issues, e.g. the cost-effectiveness or the reproducibility of the modality, are still under evaluation. However, we have presented our relevant comments in the tables. Finally, considering that the data were not eligible for synthesis of the results, we considered it inappropriate to perform a meta-analysis; therefore, we decided to present our results in a narrative manner.

## Conclusions

Varicocele seems to alter the histological architecture of the affected testicles and this phenomenon can be detected as a change in stiffness by elastography. These differences might be attributed to underlying fibrosis or atrophy. As elastography has also shown a meaningful correlation with spermatogenesis parameters, this modality could provide an in-depth assessment of the severity of VC. In infertile men who are candidates for treatment, elastography could predict the improvement in semen parameters. However, the exact merit of elastography to date remains uncertain as clinical indications still come first when dealing with VCs in infertile men. Further research should clarify the exact role of elastography in the evaluation and management of infertile men with VC.
